# Evaluation of structural and vascular changes in the choroid after uneventful phacoemulsification surgery

**DOI:** 10.22336/rjo.2023.9

**Published:** 2023

**Authors:** Mehmet İçöz

**Affiliations:** * Department of Ophthalmology, Yozgat City Hospital, Yozgat, Turkey

**Keywords:** phacoemulsification surgery, choroid, choroidal vascular index

## Abstract

**Objective:** The aim of this study was to evaluate structural and vascular changes in the choroid after phacoemulsification surgery.

**Methods:** This research comprised 50 eyes of 50 individuals who received uneventful surgical treatment for cataracts. Intraocular pressure, axial length, subfoveal choroidal thickness, and choroidal thickness in the nasal and temporal areas at 1,500-micron distance from the fovea were measured before surgery and at one and three months after surgery. At the same evaluation times, the choroidal luminal area and choroidal stromal area were calculated with the binarization method using image J software, and the choroidal vascular index (CVI) was calculated by dividing the luminal area by the total area. In addition, total surgery time and effective phacoemulsification time were recorded.

**Results:** The patients had a mean age of 64.7 ± 8.5 years. The mean total operative time was 13.9 ± 3.8 minutes, and the mean effective phacoemulsification time was 3.8 ± 2.1 minutes. The mean intraocular pressure value was 14.4 ± 3.4 mmHg before surgery, and 13.2 ± 2.9 mmHg at the first month and 13.0 ± 2.1 mmHg at the third month postoperatively. Although there was a decrease in the intraocular pressure after surgery, no significant difference was found (p>0.05 for all). The axial length measured during the first and third months postoperatively did not significantly differ from the evaluation undertaken preoperatively (p>0.05 for all). A significant increase was detected in the subfoveal, nasal, and temporal choroidal thicknesses at the first postoperative month compared to the preoperative values (p<0.05), but no significant difference was found at the third month postoperatively (p>0.05). The mean CVI was 61.6 ± 3.5% preoperatively, 65.2 ± 4.2% at the first postoperative month, and 65.9 ± 3.9% at the third postoperative month. The increase at the first and third postoperative months was significant when compared to the evaluation made preoperatively (p=0.004 and p<0.001, respectively).

**Conclusion:** Structural and vascular choroidal changes in the after-cataract surgery are important. In this study, it was observed that after uneventful phacoemulsification, the choroidal thickness increased at the first postoperative month and reached the preoperative values at the third month. It was also determined that CVI increased at the first and third postoperative months. CVI can offer an idea about whether cataract surgery is a predisposing factor in diseases involving the choroid.

**Abbreviations:** CVI = choroidal vascular index

## Introduction

Currently, phacoemulsification is one of the most preferred ophthalmologic surgery methods to increase visual acuity and among the most commonly performed intraocular operations in the treatment of cataracts [**1**]. The choroid is a tissue located between the sclera and retinal pigment epithelium, which has a vascular network rich in oxygenation and supplies the outer 1/ 3 of the retina with nutrition [**2**]. Due to this relationship, the choroid is involved in various pathologies [**3**], including choroidal neovascularization, central serous chorioretinopathy, and polypoidal choroidal vasculopathy [**4**-**6**]. Structural changes in the choroid may vary depending on many factors, such as age, refractive error, intraocular pressure, and axial length [**7**].

With its deeper penetration ability, swept-source optical coherence tomography allows imaging deeper tissues with high resolution [**8**]. This method is useful for deeply located tissues, such as the choroid, and it provides reproducible and reliable results in a non-invasive manner [**9**].

In our clinical practice, we encountered patients that did not have any improvement in the visual prognosis after cataract surgery without any detectable cause, as well as those with pathologies affecting the choroid, such as senile macular degeneration. This led us to investigate the relationship between cataract surgery and the choroid. Although there are many studies in literature evaluating choroidal thickness after cataract surgery, only few have focused on vascular changes [**10**-**12**]. Therefore, the aim of the current study was to examine structural and vascular changes in the choroid after phacoemulsification surgery. To this end, we evaluated not only choroidal thickness, but also the choroidal vascular index (CVI), which provided information on the vascular structure of the choroid. 

## Material and method

This cross-sectional observational study included patients followed up for a minimum of three months after uneventful cataract surgery at Yozgat City Hospital. Ethical approval was obtained from the local ethics committee of Ankara City Hospital (number: E1-22-3095). All patients who participated in the study provided an informed consent, and the research was undertaken in accordance with the principles of the Declaration of Helsinki. 

The study included 50 eyes of 50 patients who underwent uneventful cataract operations. The exclusion criteria were determined as cataracts other than senile cataract, glaucoma, uveitis, trauma, any previous eye surgery or ocular disease, diabetes, or additional systemic disease, and smoking history. In addition, patients with intraoperative complications, such as a radial tear in the anterior capsule, zonular weakness, posterior capsule rupture, and dropped nuclei, and those with postoperative intense or persistent corneal edema, anterior chamber reaction, wound leakage, posterior capsule opacification, and macular edema were excluded.

A detailed ophthalmological examination was performed on all participants. Visual acuity, intraocular pressure, and axial length measurements and anterior segment and fundus examinations were performed preoperatively and at the first and third months postoperatively. Preoperative cataract hardness was determined according to the lens opacities classification system (LOCS) [**13**]. Patients with grade 2-4 cataracts were included in the study, while those with hard cataracts that could affect measurement quality were excluded. Axial length measurement was performed using the IOLMaster 500 device (Carl Zeiss Meditec AG, Jena, Germany).

All operations were performed by the same surgeon (M.I.). All the patients underwent surgery with a Whitestar Signature Pro (Johnson & Johnson Vision, USA) phacoemulsification device and the same one-piece hydrophobic intraocular lens. The total operative time and effective phacoemulsification time were recorded. Postoperatively, topical antibiotics were prescribed for one week, and topical steroids for one month with gradual reduction.

Choroidal thickness was measured with a spectral-domain OCT device (OCT; Cirrus OCT; Carl Zeiss Meditec, Dublin, CA) at the same time of day (9-11 a.m.). The vertical distance from the outer border of the retinal pigment epithelium to the choroidoscleral interface was measured to obtain choroidal thickness. The measurements were conducted using sections that vertically passed through the fovea and were 1.5 mm nasal and temporal to this structure. All the measurements were evaluated twice by the same investigator (M.I.), and inconsistent measurements were excluded.

CVI was calculated using free Image J software (version 1.52a, available for public available at http://imagej.nih.gov/ij/) on the HD line images obtained with spectral-domain OCT. The dark and light pixels indicated the luminal or vascular region and the stromal or interstitial region, respectively. CVI was accepted as the ratio of the vascular area to the total circumscribed area [**14**].

In each patient, all the measurements were performed and recorded preoperatively and at the first and third postoperative months. 

The Statistical Package for the Social Sciences was used to analyze the data (version 22.0 Chicago, IL, USA). Quantitative values were expressed as mean ± standard deviation. The conformance of the data to a normal distribution was evaluated visually by histograms and statistically by the Kolmogorov-Smirnov test. The repeated measurements of a single group were analyzed with the general linear model. For statistical significance, p < 0.05 was taken as the limit.

## Results

The study included 50 eyes of 50 individuals (29 females and 21 males), who had cataracts with grade 2-3 hardness according to the LOCS classification [**13**] and underwent uneventful cataract surgery. The patients had a mean age of 64.7 ± 8.5 years. Twenty-six of the operated eyes were the right eyes and 24 were the left eyes. The total operative time was 13.9 ± 3.8 minutes, and the effective phacoemulsification time was 3.8 ± 2.1 minutes (**Table 1**).

**Table 1:** Demographic characteristics, surgical side, and phacoemulsification parameters of the patients participating in the study

Variable	Mean ± SD
Age, years	64.7 ± 8.5
Gender (female/ male)	29/ 21
Laterality (right/ left)	26/ 24
Total operative time (min)	13.9 ± 3.8
Effective phacoemulsification time (min.)	3.8 ± 2.1
SD = standard deviation, min. = minute	

The visual acuity of the patients was 0.75 ± 0.21 logMAR before surgery, and 0.11 ± 0.05 logMAR at the first month and 0.07 ± 0.02 logMAR at the third month after surgery. The intraocular pressure value was 14.4 ± 3.4 mmHg preoperatively, 13.2 ± 2.9 mmHg at the first month postoperatively, and 13.0 ± 2.1 mmHg at the third month postoperatively. Axial length was 23.8 ± 1.1 mm preoperatively, 23.7 ± 1.0 mm at the first postoperative month, and 23.5 ± 0.8 mm at the third postoperative month. 

The visual acuity of the patients was 0.75 ± 0.21 logMAR in the preoperative period, 0.11 ± 0.05 logMAR at the first month after surgery, and 0.07 ± 0.02 logMAR at the third month after surgery. The preoperative intraocular pressure was 14.4 ± 3.4 mmHg, and the postoperative intraocular pressure was 13.2 ± 2.9 mmHg at the first month and 13.0 ± 2.1 mmHg at the third month. Axial length was 23.8 ± 1.1 mm preoperatively, 23.7 ± 1.0 mm at the first month after surgery, and 23.5 ± 0.8 mm at the third month after surgery. The subfoveal choroidal thickness of the patients was 245.2 ± 40.8 μm preoperatively, 265.8 ± 35.1 μm at the first postoperative month, and 258.7 ± 31.4 μm at the third postoperative month. The nasal choroidal thickness measured at 1,500-micron distance from the fovea was 277.8 ± 47.9 μm preoperatively, 296.5 ± 32.3 μm at the first postoperative month, and 288.4 ± 29.3 μm at the third postoperative month. The temporal choroidal thickness measured at 1,500-micron distance from the fovea was 271.1 ± 42.3 μm preoperatively, 289.6 ± 35.5 μm at the first postoperative months, and 279.8 ± 30.3 μm at the third postoperative month. These values are presented in **Table 2**.

**Table 2:** Visual acuity, intraocular pressure, axial length, and choroidal thickness according to the evaluation times

	Before surgery	First month after surgery	Third month after surgery
Visual acuity (logMAR)	0.75 ± 0.21	0.11 ± 0.05	0.07 ± 0.02
Intraocular pressure (mmHg)	14.4 ± 3.4	13.2 ± 2.9	13.0 ± 2.1
Axial length (mm)	23.8 ± 1.1	23.7 ± 1.0	23.5 ± 0.8
Subfoveal choroidal thickness (μm)	245.2 ± 40.8	265.8 ± 35.1	258.7 ± 31.4
Nasal choroidal thickness at 1.5 mm from the fovea (μm)	277.8 ± 47.9	296.5 ± 32.3	288.4 ± 29.3
Temporal choroidal thickness at 1.5 mm from the fovea (μm)	271.1 ± 42.3	289.6 ± 35.5	279.8 ± 30.3
μm = micrometer, logMAR = logarithmic minimum angle of resolution			

Among the choroidal area measurements, the luminal area was 48.2 ± 5.6% preoperatively, 50.1 ± 5.2% at the first postoperative month, and 50.3 ± 4.9% at the third postoperative month. The choroidal stromal area was 28.4 ± 2.4% preoperatively, 26.2 ± 2.2% at the first postoperative month, and 26.6 ± 1.9% at the third postoperative month. CVI was 61.6 ± 3.5% preoperatively, 65.2 ± 4.2% at the first month postoperatively, and 65.9 ± 3.9% at the third month postoperatively. **Table 3** shows these values.

**Table 3:** Choroidal area and CVI values according to the evaluation times

	Before surgery	First month after surgery	Third month after surgery
Choroidal luminal area	48.2 ± 5.6	50.1 ± 5.2	50.3 ± 4.9
Choroidal stromal area	28.4 ± 2.4	26.2 ± 2.2	26.6 ± 1.9
CVI	61.6 ± 3.5	65.2 ± 4.2	65.9 ± 3.9
CVI = choroidal vascular index			

Intraocular pressure and axial length did not significantly differ in the comparison of the measurements performed at three evaluation times (p>0.05 for all). Significant increases were found in the subfoveal, nasal, and temporal choroidal thicknesses from the preoperative time to the third month after surgery (p=0.02, p=0.01, and p=0.02, respectively); however, the comparison of the preoperative and postoperative third-month measurements did not indicate any significant difference (p=0.14, p=0.25, and p=0.45, respectively). Lastly, although the increases in CVI from the preoperative period to the first and third postoperative months were significant (p=0.004 and p<0.001 respectively), the comparison of the measurements performed at the first and third months after surgery did not show any significant difference (p=0.34) (**Table 4**).

**Table 4:** Significance of changes in intraocular pressure, axial length, choroidal thickness, and CVI

Parameter	P value		
	Preop. vs. postop. first month	Preop. vs. postop. third month	Postop. first vs. third month
Intraocular pressure	0.09	0.08	0.56
Axial length	0.41	0.56	0.54
Subfoveal choroidal thickness	0.02	0.14	0.21
Nasal CT	0.01	0.25	0.32
Temporal CT	0.02	0.45	0.56
CVI	0.004	<0.001	0.34
preop. = preoperative, postop. = postoperative, CT = choroidal thickness, CVI = choroidal vascular index			

## Discussion

In literature, there are many studies on structural changes in the retina and choroid following cataract operations. Moreover, there are many studies on structural retinal and choroidal changes following cataract operations [**15**-**17**]. With the introduction of OCT, knowledge on this subject is increasing day by day. The detection of changes in the choroid and retina using OCT provides guidance in diagnosing and treating senile macular degeneration, spectrum of pachychoroid disorders, and vascular pathologies [**15**,**16**]. After uneventful cataract surgery, conditions such as pseudophakic cystoid macular edema, aggravation of existing retinal pathologies, or lack of the expected increase in visual acuity may occur [**17**]. Therefore, in pathologies that cause a decrease in visual acuity and in patients that do not have increased visual acuity after cataract surgery, there may be a mechanism involving the retina and choroid. In this study, we aimed to evaluate possible structural and vascular changes that could be such a mechanism.

Despite the availability of many studies exploring the relationship between cataract surgery and choroidal thickness, it is not possible to compare these studies. Choroidal thickness alone may vary depending on diurnal rhythm, age, refractive error, and presence of systemic diseases, or it may be affected by multiple factors, such as the surgeon performing the cataract surgery, type of surgery, and device and parameters used. Therefore, in the current study, all the operations were performed by a single physician using the same device and phacoemulsification parameters. Choroidal measurements were also undertaken with the same device and method.

When studies evaluating the relationship between choroidal thickness and cataract surgery were examined, Noda et al. found that choroidal thickness increased at the first postoperative month, while Falcão et al. detected that this thickness did not differ at the first postoperative week and first month compared to the preoperative period [**18**,**19**]. Bayhan et al. evaluated choroidal thickness at first month after surgery in 38 patients and reported a significant increase [**20**]. Pierru et al. determined that the subfoveal choroidal thickness increased according to the postoperative first-week and first-month evaluations, but returned to the basal levels at the third month [**21**]. In these studies, it was considered that inflammation in the anterior segment, caused by cataract surgery, also affected the posterior segment as a common mechanism [**18**-**21**]. It is known that proinflammatory cytokines, free radicals, and prostaglandins play a role in inflammation. Surgical trauma and impaired blood-aqueous barrier also trigger the inflammatory cascade in the posterior segment [**22**-**24**]. An animal study showed that gene expressions inducing the inflammatory response increase with surgical stress [**23**]. In the same study, it was reported that proinflammatory cytokines, such as interleukin-1β, increased in the choroid and inner retinal layers of the eyes that underwent surgery [**23**]. In the current study, it was observed that the choroidal thickness measured from three points increased at the first postoperative month and approached the basal level at the third month. A possible reason for this increase in the early period may be that the inflammatory mediators generated in the anterior segment pass to the posterior segment and affect the thickness of the choroidal structures.

The increase in thickness in retinal layers after cataract surgery is related to intraocular pressure fluctuation and exposure to microscope light, and ultrasound power during surgery [**25**]. However, the lack of a clear mechanism that can explain the change in choroidal thickness has led researchers to continue to conduct studies on this subject. It has been determined that after a cataract operation, choroidal thickness increases by 5-30 µm on average, starting on the third day after surgery at the earliest and returning to preoperative values at the third month [**11**]. It has also been suggested that a longer lasting increase in thickness may be associated with a short axial length, low intraocular pressure, high basal thickness, and younger age [**26**]. In the current study, although the intraocular pressure values decreased at the postoperative visits, the intraocular pressure and axial length measurements did not indicate a significant difference between the three measurement times.

In most studies, choroidal thickness has been reported to increase after cataract surgery, but there is a need for further research to investigate structures that may be responsible for this increase in thickness. Therefore, in our study, we also evaluated CVI as a current parameter that provides information on the vascular or stromal tissue of the structure responsible for the increase in thickness.

It is considered that, in contrast to choroidal thickness defined by Agrawal et al., CVI is not dependent on multiple factors and can therefore be in a more stable range; therefore, the use of CVI together with choroidal thickness can offer more information concerning the structural and vascular changes that may occur in the choroid [**27**]. It has been reported that CVI can be used for early diagnosis and evaluation of treatment response in central serous chorioretinopathy and for diagnosis in atrophic macular degeneration [**28**,**29**]. Both pathologies are diseases affecting the choroid [**28**,**29**]. Stress factor is known to be included in the etiology of central serous chorioretinopathy [**28**]. Cataract surgery is also an intraocular operation, in which inflammatory mediators are likely to pass from the anterior to posterior chamber and create surgical stress. While studies conducted in the past years reported that atrophic macular degeneration increased after cataract surgery, recent research indicates that this surgery does not influence the progression of atrophic macular degeneration [**30**,**31**]. 

In a three-month follow-up of 36 patients, Chen et al. reported that CVI increased at all the control visits after surgery, and only choroidal thickness had a significant relationship with this increase [**32**]. However, in that study, the effective phacoemulsification time and total operative time were not evaluated, and the thickness of the choroid was only evaluated in the subfoveal region [**32**]. To the contrary, in the current study, we also measured choroidal thickness in the nasal and temporal areas at 1,500 microns from the fovea and evaluated the effective phacoemulsification and total operative times.

Yao et al. evaluated CVI in patients with diabetic cataracts and reported a significant increase after surgery [**1**]. In contrast to an earlier study suggesting that changes in intraocular pressure after cataract surgery might lead to changes in choroidal thickness [**20**], Yao et al. found no significant postoperative difference in intraocular pressure, despite a decrease [**1**]. Similarly, in the current study, there was a decrease in intraocular pressure at the first and third postoperative month evaluations, but this was not statistically significant with respect to the measurement made before surgery.

Apolloni et al. performed the comparison of the choroidal thickness and CVI values after Femto laser-assisted and conventional phacoemulsification surgery and reported an increase in both values after conventional surgery [**33**].

An increase in CVI was observed in the evaluation we performed at the third month after cataract surgery, while the choroidal thickness values approached preoperative values. Due to being dependent on many factors, choroidal thickness measurements may be labile, while CVI is affected by less factors. Dilatation in choroidal vessels regains a structure similar to its previous state, but the ratio of vascular and stromal structures within the tissue may vary, which can explain the increase in the CVI value.

Among the limitations of the study, first, CVI is a two-dimensional evaluation, and we calculated this parameter in a 1,500-micron area; therefore, it may not reflect the whole choroid. There is a need for histological studies to provide more information on the luminal and stromal areas of the choroid. Second, our follow-up period was three months. Although the increase in choroidal thickness and return to the basal level after surgery were consistent with literature, the increase in CVI continued. There is a need for studies that have a longer-term follow-up to elucidate the course of this increase. Third, more detailed information on this subject can be obtained in multi-center studies to be conducted with a larger number of patients.

## Conclusion

In conclusion, cataract surgery may be a predisposing factor in various diseases involving the choroid directly or indirectly. Therefore, structural and vascular changes in the choroid after cataract surgery are important. In this study, it was observed that choroidal thickness increased at the first postoperative month after uneventful phacoemulsification and approached the preoperative values at the third postoperative month. Considering the significant increase in the CVI value at the first- and third-month evaluations performed after surgery, this parameter can be used to evaluate the relationship between cataract surgery and diseases involving the choroid.

**Conflict of Interest statement**

The authors declare that they have no conflicts of interest or affiliation with any organization or person that has a financial or non-financial interest in the topic or materials mentioned in this work.

**Informed Consent and Human and Animal Rights statement**

All participants provided an informed consent. 

**Authorization for the use of human subjects**

The study involving human subjects conforms with all applicable national rules and institutional policies and the tenets of the Declaration of Helsinki. Prior to the study, approval was obtained from the local ethics committee of Ankara City Hospital, Turkey (no: E1-22-3095).

**Acknowledgements**

No acknowledgment to declare. 

**Sources of Funding**

This study did not receive any grants from governmental, commercial, or non-profit funding organizations.

**Disclosure (s)**

None. 
